# Setting the Priorities for LGBT+ Research and Intervention Effort in Malaysia Through Community Voices: A Brief Report

**DOI:** 10.1177/2752535X241273831

**Published:** 2024-08-09

**Authors:** Kyle Tan

**Affiliations:** 1 Faculty of Māori and Indigenous Studies, 3717 University of Waikato, Hamilton, New Zealand

**Keywords:** LGBT, LGBTQ, gay, transgender, Malaysia, Southeast Asia, criminalization

## Abstract

Internationally, there is a growing acceptance of gender and sexuality diversity and acknowledgment of LGBT + identities as health determinants. However, caution is warranted when applying research and intervention priorities from Global North countries to regions where LGBT + identities remain criminalized. In 2024, Malaysia maintains legal stances persecuting LGBT + individuals and shows no intent to address this human rights issue. This study offers an overview of pivotal issues identified by LGBT + communities in Malaysia that urgently require attention and resolution. Data were employed from a large-scale community-based survey: the KAMI Survey that recruited LGBT + participants in Malaysia in late 2023 and descriptive analyses were conducted on the responses of 637 participants (mean age = 27.75). Results revealed key issues deemed ‘very important’ to address by participants comprised HIV/AIDS, training for healthcare providers, police mistreatment, and discrimination, with more than 80% reporting each of these. When prompted to select a single issue for urgent resolution, three-fifths (61.0%) prioritized ‘criminalizing laws affecting LGBT + individuals’. Echoing prolonged advocacy by local LGBT + community organizations, the author emphasizes the need for collective allyship across stakeholders to develop evidence-based practices and policies to address the concerns articulated in this paper.

## Introduction

Globally, there is an increasingly recognized consensus of LGBT + identities as a determinant of health.^1,2^ Yet, the operation and costs of systemic and institutional cisheterosexism that criminalize, pathologizes, and marginalizes individuals who do not conform to cisgender and heterosexual expectations remain under-explored in Southeast Asia countries.^3^ Within the scant LGBT + studies in Malaysia, a faction of researchers with malicious intent exists to perpetuate cisheterosexist views and spread disinformation about LGBT + communities.^4^ Existing research on this minoritized population also tends to follow a top-down approach, often conducted *on* LGBT + communities rather than *in collaboration with* or *led by* the communities themselves. The Malaysian Government seldom prioritizes equity-based intervention efforts for LGBT individuals, except in the context of plans to address HIV transmission among at-risk groups which occasionally accounts for MSM (men who have sex with men).^5^ Consequently, existing research involving LGBT + participants in Malaysia has predominantly centered on HIV prevention.^5^ This narrow focus comes at the expense of addressing structural barriers that hinder the affirmation of LGBT + identities, such as criminalizing laws targeting LGBT + individuals, discriminatory policies, and the endorsement of conversion efforts by politicians and religious leaders.^6,7^

The specific challenges encountered by LGBT + Malaysians are intricately tied to the repressive climate upheld by both secular and non-secular laws prosecuting this population.^8^ As a former British colony, Malaysia inherited the Section 377 penal code as the colonizer’s attempt to dictate behavioral norms (e.g., prohibiting same-sex relationships) to safeguard themselves against moral lapses. Despite its repeal in several countries such as India and Singapore, Malaysia persists in retaining this penal code which contradicts the international human rights standard of LGBT + individuals (see for example, the Yogyakarta Principles).^9^ Islam is the official religion of Malaysia and Sharia law applies only to the Muslims of the country (comprising about 63.5% of the population).^8^ Sharia law explicitly criminalizes same-sex practices and gender diverse people who engage in musahaqah (sexual relationships between women), tasyabbuh (expressing gender different from their assigned sex at birth), and liwat (same-sex sexual intercourse).^8^ Compared to Global North countries that have celebrated milestones in advancing LGBT + equity, LGBT + Malaysians continue to endure heightened scrutiny from law enforcement, become subjects of political manipulation, and face constraints in openly expressing their identity and orientation.^10^

Despite the overlaps in challenges faced by LGBT + individuals across different countries (e.g., barriers to accessing culturally safe healthcare and tackling disinformation targeting LGBT + people), there is a critical necessity to delineate research and intervention priorities specifically for LGBT + Malaysians. Doing so would enable the identification of strategic plans geared towards instigating change and breaking the cycle of ‘no funding for initiating LGBT + research - no evidence generated - no problem found – LGBT + communities not identified as a priority for intervention’. To amplify community voices on the key concerns affecting LGBT + individuals in Malaysia, we utilized data from one of the largest community-based survey of LGBT + participants conducted in Malaysia to date: the Kami Survey.

## Method

Led by a team of queer-identified researchers and allies, the Kami Survey was developed in consultation with LGBT + community organizations in Malaysia. Its primary objective is to gather empirical data on the extent of stigma and the barriers faced by Malaysian LGBT + individuals in accessing social determinants of health (e.g., healthcare and family support) and the mental health inequities faced by this population. The survey was open for responses from October 1st to December 16th, 2023. It was accessible in both English and Malay languages and participants provided their consent by completing either version of the survey. The collaboration with LGBT + community organizations including JEJAKA, Justice for Sisters, PLUHO, and SEED Foundation, was essential as they offered platforms such as social media and in-person activities to engage the survey’s target groups. Snowball sampling was also utilized by encouraging participants to spread words about the survey within their networks, and the first 250 participants were offered a RM 10 voucher. Further details about the survey method can be viewed from another published report.^11^

Participants were presented with a list of issues and asked to rate the urgency of addressing each (see Table 1). This adapted question from the US Trans Survey^12^ was tailored to fit the Malaysian context through consultation with LGBT + community organizations. Following this, participants were prompted to select the most important issue for immediate attention. Descriptive analyses were carried out in SPSS v29.

**Table 1.** Demographic Information of KAMI Survey participants who Completed the Section on Important Issue to Address for LGBT + People in Malaysia.

	*n* (%)
**Age** (mean; SD)	27.75 (6.93)
Gender groups^a^	
Cis man	286 (44.9)
Cis woman	179 (28.1)
Non-binary	78 (12.2)
Trans man	35 (5.5)
Gender questioning	32 (5.0)
Trans woman	27 (4.2)
Sexual orientation	
Gay and lesbian	354 (55.8)
Bisexual and pansexual	192 (30.3)
Queer and questioning	47 (7.5)
Asexual	27 (4.3)
Heterosexual	14 (2.2)
Ethnic groups^b^	
Chinese	291 (45.7)
Malay and Indigenous peoples of West Malaysia (Orang Asli)	291 (29.2)
Indian	88 (13.8)
Bumiputera (natives) of Sabah and Sarawak	39 (6.1)
Others	33 (5.2)
Citizenship status	
Malaysian citizen	616 (97.2)
Permanent resident and non-citizen	18 (2.3)
Average monthly household income (MYR)	
Less than 1000	54 (9.3)
1000 to 1999	48 (8.2)
2000 to 3999	165 (28.3)
4000 to 5999	112 (19.2)
6000 to 7999	54 (9.3)
8000 to 9999	32 (5.5)
10,000 and above	118 (20.2)

^a^Gender groups were categorized using the two-steps method (Fraser, 2018).

^b^Ethnic groups were categorized in accordance with the Malaysian National Health and Morbidity Survey (National Institutes of Health, 2019).

MYR = Malaysian Ringgit; SD = standard deviation.

## Results

Table 1 details the demographic profile of participants. More than two-thirds were young adults below 30 years old (67.7%), with the age of sample ranged from 18 to 61. More than two-thirds were cis men or women (73.0%) and one-fifth identified as trans or non-binary (21.9%). More than half identified as gay or lesbian (55.8%). A majority held a Malaysian citizenship (97.2%) with the three largest ethnic groups represented were Chinese (45.7%), Malay and Orang Asli (29.2%) and Indian (13.8%). Over two-thirds resided primarily in Selangor (45.3%) or Kuala Lumpur (29.2%). Not all participants responded to all questions due to attrition and missing data were removed using listwise deletion.

The top four issues that participants rated as ‘very important’ to address were HIV/AIDS, training for healthcare providers, police mistreatment, and discrimination, with more than four-fifths reporting each of these (see Table 2). Over seven-tenths identified issues including criminalizing laws, employment challenges, housing insecurity, conversion efforts, and education for family members as ‘very important’ concerns. Participants were given an open-text box to write-in additional issues of importance beyond the provided list; these included legal gender recognition, hate speech laws, access to education, community building, and equitable care approaches for LGBT + individuals. Table 3 displays the priority order of issues as participants selected a single concern for urgent resolution. More than three-fifths highlighted ‘criminalizing laws affecting LGBT + individuals’ as the primary concern, followed by ‘discrimination’ that was endorsed by over one-tenth of participants.

**Table 2.** The Degree of Importance to Address Each Issue That Affects LGBT + People in Malaysia Urgently.

	Very important, *n* (%)	Important, *n* (%)	Not very important, *n* (%)
HIV/AIDS (*n* = 635)	545 (85.8)	81 (12.8)	9 (1.4)
Training healthcare providers about LGBT+health (*n* = 635)	544 (85.7)	89 (14.0)	2 (0.3)
Police mistreatment of LGBT+people (*n* = 633)	540 (85.3)	77 (12.2)	16 (2.5)
Discrimination against LGBT+people (*n* = 635)	535 (84.3)	83 (13.1)	17 (2.7)
Criminalizing laws for LGBT+people (e.g., Section 377A and Sharia law) (*n* = 636)	502 (78.9)	98 (15.4)	36 (5.7)
Employment (*n* = 636)	483 (75.9)	122 (19.2)	31 (4.9)
Housing and homelessness (*n* = 635)	460 (72.4)	142 (22.5)	32 (5.0)
Conversion therapy (e.g., efforts to change your LGBT+identity) (*n* = 635)	459 (72.3)	96 (15.1)	80 (12.6)
Education for family members about LGBT+identity (*n* = 633)	443 (70.0)	169 (26.7)	21 (3.3)
Poverty (*n* = 634)	442 (69.7)	142 (22.4)	50 (7.9)
Easier and safe access to gender-affirming care (e.g., hormones) (*n* = 636)	417 (65.6)	186 (29.2)	33 (5.2)
Parenting and adoption rights for LGBT+families (*n* = 632)	396 (62.7)	176 (27.8)	60 (9.5)
Marriage rights for LGBT+people (*n* = 636)	289 (45.4)	214 (33.6)	133 (20.9)

**Table 3.** The Most Important Issue to Address for LGBT + People in Malaysia (*n* = 616).

	*n* (%)
Criminalizing laws for LGBT+people (e.g., Section 377A and Sharia law)	376 (61.0)
Discrimination against LGBT+people	71 (11.5)
HIV/AIDS	44 (7.1)
Marriage rights for LGBT+people	27 (4.4)
Conversion therapy (e.g., efforts to change your LGBT+identity)	27 (4.4)
Police mistreatment of LGBT+people	21 (3.4)
Training health professionals (eg, doctor and psychiatrist) about LGBT+health	12 (1.9)
Employment	12 (1.9)
Education for family members about LGBT+identity	12 (1.9)
Easier and safe access to gender-affirming care (e.g., hormones)	6 (1.0)
Parenting and adoption rights for LGBT+families	5 (0.8)
Housing and homelessness	2 (0.3)
Poverty	1 (0.1)

## Discussion

This study presents a novel insight on the intervention needs of LGBT + individuals within a societal framework where the criminalization of LGBT + identities persists. In contrast to nations advocating for marriage equality, parenting and adoption rights, and substantiating associated mental health advantages,^13^ these are regarded as ‘privileges’ and are relegated to lower priority status by LGBT + individuals in Malaysia. The disparity between the top issue rated as ‘very important’ to address (i.e., HIV/AIDS) and subsequent concern on criminalizing laws may stem from participants’ limited confidence in the change in institutional status quo. This skepticism could be attributed to successive governments that have shown minimal commitment to enhancing LGBT + equity and, at times, pathologize LGBT + identities to appease religious conservatives and scapegoat LGBT + individuals for their poor social and health outcomes.^14^ Nevertheless, an overwhelming majority of participants identified challenging the criminalization of LGBT + individuals in Malaysia as the utmost priority. This finding mirrors the ongoing struggle of LGBT + communities to attain fundamental human rights.

There are crucial lessons that Malaysia could learn from its neighbour, Singapore, which officially repealed Section 377 penal code in 2023.^15^ Scholars viewed this decision as strategic as the Singapore Government sought to promote the country’s image as an “open, diversified global financial center”, with the retention of the penal code seen as antithetical to the global trend of supporting LGBT + equity.^15^ While LGBT + communities in Singapore who had advocated for identity-specific decriminalization celebrated the legal reform, much progress is still needed to advance LGBT + rights in Singapore.

The response from most survey participants echoes the extensive groundwork laid by LGBT + organizations over many years to address criminalizing laws targeting LGBT + people. In 2021, multiple grassroot LGBT + community organizations in Malaysia recommended the Government to “repeal laws that criminalize LGBT persons based on sexual orientation, gender identity and gender expression given the magnitude of its impact, especially on access to redress and remedies, ability to live with dignity, privacy and safety, among others” (p.33).^10^ The escalating state-sponsored anti-LGBT efforts were again highlighted during the Universal Periodic Review (UPR) hosted by the United Nations Human Rights Council, where Justice for Sisters^14^ reiterated the call for the Government to promptly review regulations (including secular and Sharia laws) to ensure compliance with international human rights standards of LGBT + individuals. The imperative to decriminalize and depathologize LGBT + identities cannot solely rest upon LGBT + community organizations and members. Instead, it requires the concerted mobilization of collective allyship among health professionals, policymakers, educators, and researchers to bolster the evidence base essential for shaping and implementing equitable practices and policies addressing key concerns outlined in this paper.

As the survey relied on convenience sampling for recruitment, caution should be exercised when generalizing the findings. The sample’s demographics deviated from reflecting the general population,^16^ with an overrepresentation of Chinese, Indian, and younger participants. The survey’s recruitment method also favored LGBT + individuals with internet access and familiarity with services of LGBT + organizations. Further, the survey item about criminalizing laws lacked differentiation which may have diluted the more pronounced impact of the enactment of Sharia law on Muslim participants. The lower number of responses to the follow-up question about ‘the most important issue to address’ could be due to some participants facing difficulty in navigating the drop-down option. Despite these limitations, the KAMI Survey stands as the largest effort to capture the voices of Malaysia’s LGBT + individuals, nearly tripling the size of previous studies of a similar nature.^17^

## Data Availability

The data that support the findings of this study are available on request from the corresponding author. The data are not publicly available due to privacy or ethical restrictions.
